# ATM Kinase Inhibition Preferentially Sensitises PTEN-Deficient Prostate Tumour Cells to Ionising Radiation

**DOI:** 10.3390/cancers13010079

**Published:** 2020-12-30

**Authors:** Conor Hanna, Victoria L. Dunne, Steven M. Walker, Karl T. Butterworth, Nuala McCabe, David J. J. Waugh, Richard D. Kennedy, Kevin M. Prise

**Affiliations:** 1 Patrick G Johnston Centre for Cancer Research, Queen’s University Belfast, Belfast BT9 7AE, UK; channa28@qub.ac.uk (C.H.); V.Dunne@qub.ac.uk (V.L.D.); s.walker@qub.ac.uk (S.M.W.); k.butterworth@qub.ac.uk (K.T.B.); nuala.mccabe@qub.ac.uk (N.M.); 2 Almac Diagnostics, Craigavon BT63 5QD, UK; 3 School of Biomedical Sciences, Faculty of Health, Queensland University of Technology, Brisbane 4000, Australia; d6.waugh@qut.edu.au

**Keywords:** prostate cancer, PTEN, ATM, ionising radiation, DNA damage, ROS

## Abstract

**Simple Summary:**

Prostate cancer is the most frequently diagnosed cancer in men. Despite the importance of radical radiotherapy for the management of this disease, recurrence remains a challenge. PTEN is a tumour suppressor that is frequently inactivated in advanced prostate cancer and has been associated with relapse following radiotherapy. The present study shows that the role of PTEN in response to ionizing radiation is complex. Furthermore, it demonstrates that in the absence of PTEN, an increased response to combined treatment using radiotherapy and the ATM inhibitor KU-60019 can be observed. Our findings provide a strong rationale for evaluating loss of PTEN in prostate cancer as a therapeutic target for ATM inhibitor in combination with radiotherapy in the clinical setting.

**Abstract:**

Radical radiotherapy, often in combination with hormone ablation, is a safe and effective treatment option for localised or locally-advanced prostate cancer. However, up to 30% of patients with locally advanced PCa will go on to develop biochemical failure, within 5 years, following initial radiotherapy. Improving radiotherapy response is clinically important since patients exhibiting biochemical failure develop castrate-resistant metastatic disease for which there is no curative therapy and median survival is 8–18 months. The aim of this research was to determine if loss of PTEN (highly prevalent in advanced prostate cancer) is a novel therapeutic target in the treatment of advanced prostate cancer. Previous work has demonstrated PTEN-deficient cells are sensitised to inhibitors of ATM, a key regulator in the response to DSBs. Here, we have shown the role of PTEN in cellular response to IR was both complex and context-dependent. Secondly, we have confirmed ATM inhibition in PTEN-depleted cell models, enhances ionising radiation-induced cell killing with minimal toxicity to normal prostate RWPE-1 cells. Furthermore, combined treatment significantly inhibited PTEN-deficient tumour growth compared to PTEN-expressing counterparts, with minimal toxicity observed. We have further shown PTEN loss is accompanied by increased endogenous levels of ROS and DNA damage. Taken together, these findings provide pre-clinical data for future clinical evaluation of ATM inhibitors as a neoadjuvant/adjuvant in combination with radiation therapy in prostate cancer patients harbouring PTEN mutations.

## 1. Introduction

External beam radiotherapy is a well-established treatment option for locally advanced prostate cancer (PCa). Despite the high rates of local tumour control, radioresistance is observed in approximately 20–30% of localised PCa patients following initial radiotherapy, leading to cancer-related mortality in up to 27% of patients within 5 years [1]. Improving radiotherapy response is clinically important since patients exhibiting biochemical failure develop castrate-resistant metastatic disease for which there is no curative therapy and median survival is 8–18 months.

There is a significant clinical need to identify robust tissue biomarkers that will help predict a patient’s outcome following radiotherapy. Importantly, such biomarkers may help achieve better tumour response in high-risk patients by selecting patients for more effective personalised treatment plans combining molecularly targeted drugs. The tumour suppressor phosphatase and tensin homolog (PTEN) is frequently inactivated in approximately 60% of advanced PCa and is widely implicated in the progression to metastatic castrate-resistant PCa [2]. PTEN loss has further been shown to be a prognostic factor for relapse following radiotherapy in locally advanced PCa [3]. Similarly, AKT hyperactivation, a result of PTEN loss and deregulation of the PI3K/AKT pathway, correlates with PCa progression and poor clinical outcome [4]. PTEN loss is reported to confer impaired DNA damage repair and genomic instability [5,6]. In pre-clinical studies, PTEN loss in prostate adenocarcinoma PC-3 cells was shown to be synthetically lethal to treatment with poly (ADP-ribose) polymerase (PARP) inhibitors, as a result of impaired transcriptional regulation of Rad51, a key protein involved in homologous recombination (HR) repair of DNA double-strand breaks (DSB) [7,8]. This association between PTEN loss and PARP inhibitor sensitivity was not observed however in a panel of prostate cancer cell lines [9]. A previous study from within our group identified PTEN as synthetically lethal with ATM deficiency in an siRNA screen to identify novel genes whose knockdown confers selective sensitivity to ATM loss [10]. ATM is a key sensory protein involved in regulating the DNA damage response, becoming activated by the formation of DSBs during all phases of the cell cycle. It is also independently activated by oxidative stress, with ATM-deficient cells more susceptible to oxidative-stress inducing agents [11]. PTEN-deficient in vitro cell and in vivo tumour models were shown to be selectively sensitised to treatment with ATM inhibitors. Increased endogenous ATM activation was observed in PTEN-deficient cells, resulting from high levels of oxidative stress, leading to elevated levels of DSBs. Additionally, sensitisation of PTEN-deficient cells was found to be independent of a defect in Rad51 expression.

In the present study, we aimed to characterise the role of PTEN, given its major role in relapse following radiotherapy, in the response to ionising radiation (IR) in several isogenic models. Using this approach, we tested the ability of the ATM inhibitor KU-60019 to enhance sensitivity to IR in the absence of PTEN using in vitro and in vivo models. Moreover, we aimed to elucidate the molecular mechanisms involved in mediating these responses to provide a rationale for the development of future combinations.

## 2. Results

### 2.1. The Role of PTEN in the Survival Response to Ionising Radiation Is Cell-Type Dependent

**Figure 1:**
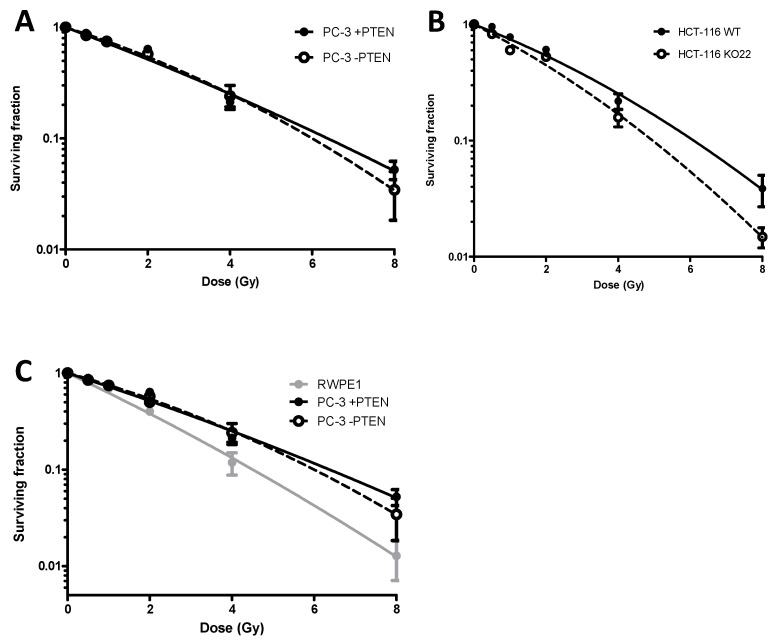
Clonogenic survival curves comparing radiosensitivity of (**A**) PC-3, (**B**) HCT-116, +PTEN (●) and −PTEN (■) isogenic human tumour cell models and (**C**) RWPE-1 human cell model. Cells were treated with a single radiation dose of 0.5, 1, 2, 4 or 8 Gy. Experiments were performed in triplicate on at least three independent occasions. Error bars represent ± standard error of the mean (SEM). The radiation Sensitizer Enhancement Ratio (SER) were calculated from the mean survival fractions at 4 Gy.

Clonogenic survival data for the PC-3 (Figure 1A) and HCT-116 (Figure 1B) isogenic models were obtained following exposure to a single dose of 0.5, 1, 2, 4, or 8 Gy, delivered as a uniform radiation field. The α and β radiobiological parameters for each of the curves in Figure 1, in addition to the surviving fraction at 2 Gy (SF2) and 4 Gy (SF4) values are summarised in Supplementary Table S1. The radiation sensitisation enhancement ratio (SER) described in the methods and summarised in Supplementary Table S2, was used to determine the effect of PTEN expression on cell radiosensitivity.

In the PC-3 cell model (Figure 1A), there was a non-significant increase in radiosensitivity in the wild-types, absent of PTEN, SER = 0.92. In the HCT-116 cells (Figure 1B), the SER was 0.68, indicating loss of PTEN significantly sensitised these cells to IR. The RWPE1 cell line was shown to be the most radiosensitive of the cell lines investigated with 13% survival at 4 Gy (Figure 1C).

### 2.2. ATM Inhibitor KU-60019 Potentiates Radiation-Induced Killing of PTEN-Deficient Cells

**Figure 2:**
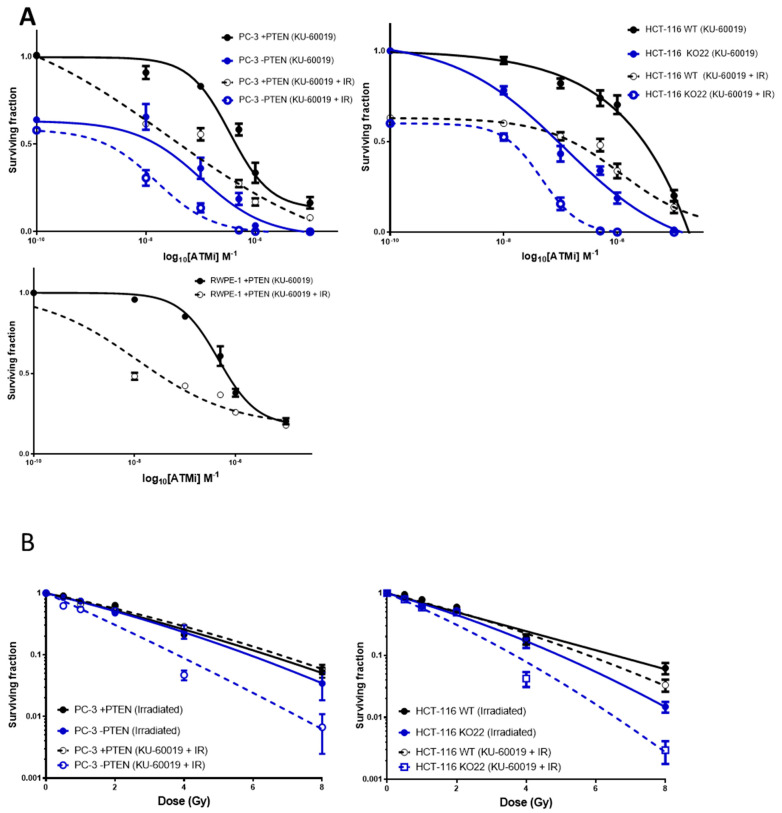
Impact of PTEN status on the survival response to combinations with radiation and KU-60019. (**A**) Dose response curves PC-3 and HCT-116 PTEN isogenic and RWPE-1 normal prostate cell models following treatment with KU-60019 alone (●, ■) or in combination with radiation (○, □). In the combined treatment groups, cells received 2 Gy ionising radiation, 1 h immediately following KU-60019 treatment. (**B**) Clonogenic survival curves of PC-3 and HCT-116 PTEN isogenic cell models following radiation (●, ■) or combined KU-60019 [LC_50_ PTEN deficient cells] + radiation treatment (○, □). LC_50_ concentrations for PTEN deficient PC-3 and HCT-116 cell lines were 21.3 nM and 137 nM respectively. Experiments were performed in triplicate on at least three independent occasions. Error bars represent ± SEM.

KU-60019 was assessed as a potential radiosensitiser of PTEN-deficient cells (Figure 2). The impact of KU-60019 depends on PTEN status in prostate models as DU145 cells had a lower RER (1.28) in comparison to LnCAP and 22RV1 cells (15.72 and 2.975 respectively) (Supplementary Figure S1 and [10]). We then confirmed this using inducible and isogenic models. Dose response curves (Figure 2A) were produced for clonogenic survival data following treatment of cells with a clinically relevant 2 Gy dose of ionising radiation (IR), 1 h immediately after treatment with KU-60019. To determine the degree of interaction between KU-60019 and IR as a combined therapy in these cell models, synergy calculations were performed and the combination indices (±1.96 SD) plotted against fraction of cells affected (1- SF) (Supplementary Figure S2). Combination index (CI) values <0.9 indicate a synergistic interaction between agents, values of 0.9–1.0 suggest additive interaction, whereas values >1.0 indicate antagonistic interactions.

In each of the isogenic models investigated, antagonistic interaction values were produced in both cells with and without PTEN at lower treatment concentrations. As the fraction of cells affected increased (treatment concentrations increased), much greater synergism was observed in PC-3 and HCT-116 PTEN-deficient cells compared to cells expressing PTEN. In the normal prostate RWPE-1 cell line, an antagonistic interaction was observed. Supplementary Table S3 shows combination index values for each cell type treated with 2 Gy + 1 µM KU-60019.

The radiation enhancement ratio (RER), obtained by dividing the mean SF for KU-60019 [1 µM] by the mean SF for KU-60019 + IR [1 µM + 2 Gy], was also used as a determinant of radiosensitisation (Supplementary Table S3). KU-60019 appears to be a potent radiosensitiser of both PTEN expressing and deficient cells but significantly greater radiosensitisation was observed in a PTEN-deficient context in each of the cell models. In the HCT-116s, RER was 9.2 times greater in PTEN-deficient cells vs. PTEN expressing cells (19 vs. 2.02).

Due to the potent radiosensitisation of KU-60019, it was desirable to improve the targeted sensitisation of the PTEN-deficient cells. Both PC-3 and HCT-116 PTEN isogenic models were pre-treated with KU-60019 [LC_50_ of PTEN-deficient cells] 1 h prior to treatment with a single IR dose of 0.5, 1, 2, 4, or 8 Gy and survival analysed by clonogenic assay (Figure 2B). LC_50_ concentrations for PTEN-deficient PC-3 and HCT-116 cell lines were 21.3 nM and 137 nM respectively. The dose enhancement ratio (DER) described in the methods showed there was little to no dose enhancement of PC-3 (DER = 0.93) or HCT-116 PTEN expressing cells (DER = 1.05) yet the PTEN-deficient LC_50_ of KU-60019 elicited a DER of 1.64 and 1.39 in PC-3 and HCT-116 cells respectively (Supplementary Table S4).

### 2.3. ATM Inhibition in Combination with IR Induces G2/M Arrest and Apoptosis in PTEN-Deficient Cells

Analysis of cell cycle distribution in the PTEN isogenic models following treatment with KU-60019 and IR was investigated due to the previously reported role for PTEN in check-point regulation [12,13,14], a function that could explain PTEN-deficient cell sensitivity to this combination treatment.

**Figure 3:**
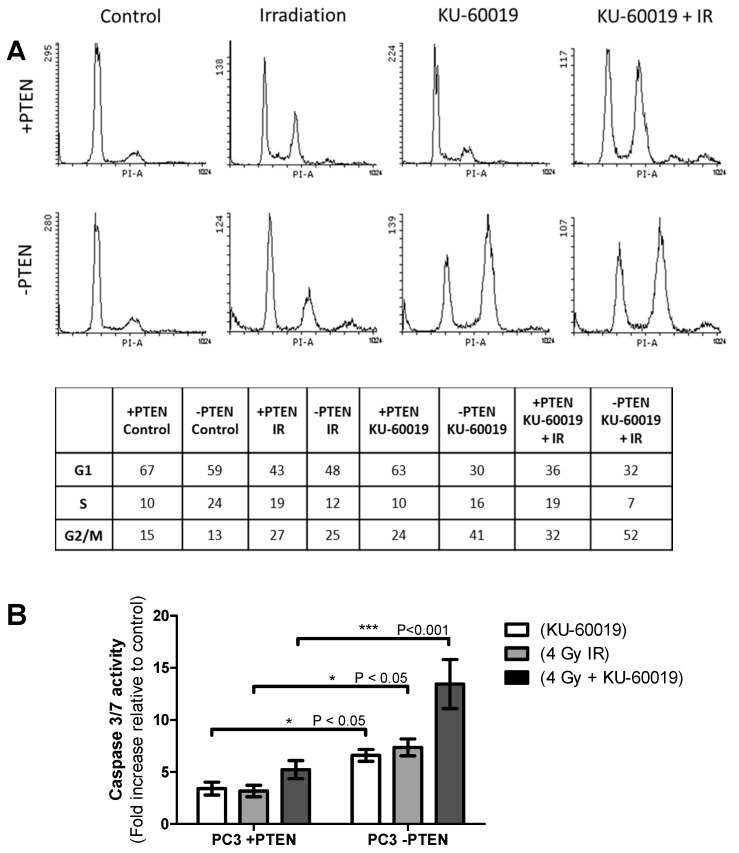
Changes in cell cycle distribution and caspase activity and its dependency on PTEN status and the combination of radiation with KU-60019. (**A**) Cell-cycle analysis using Propidium iodide (PI) staining and detection by flow cytometry of PC-3 +PTEN and −PTEN cell models after treatment with 1μM KU-60019 and 2 Gy IR, as single modalities and in combination. Analyses of cell cycle distributions 48 h post-treatment are shown. (**B**) Caspase 3/7 activity in PC-3 PTEN isogenic cell models following treatment with 1μM KU-60019 and 4 Gy IR alone or in combination. Activity was measured using a Caspase-Glo 3/7 assay kit 48 h post-treatment. Differences between two groups were compared by using a two-sample student’s *t*-test (one asterisk (*), *p* < 0.05; three asterisks (***), *p* < 0.001).

Cell cycle distribution was analysed in the PC-3 PTEN isogenic model 48 h following treatment with 1 µM KU-60019 and 2 Gy IR alone and in combination (Figure 3A). HCT-116 data is shown in Supplementary Figure S3A.

In the PC-3 cell line, irradiation led to an increase in G2/M (4n) populations and reduced G1 (2n) phase cells, independent of PTEN status. In the HCT-116 cells, however, there was a non-statistically significant increase in G2/M cell cycle arrest in PTEN-deficient cells compared to the PTEN wildtype cells (36% vs. 27%, *p* = 0.08). Exposure to KU-60019 similarly increased the proportion of cells in G2/M but this increase was significantly greater in PTEN-deficient PC-3 cells compared to PTEN expressing PC-3 cells (41% vs. 24%, *p* = 0.02). Similarly, in the HCT-116 PTEN lacking cells, there was a significant increase in the observed G2/M population compared to PTEN wildtype cells (39% vs. 27%, *p* = 0.03).

In each cell line, combination treatment with KU-60019 + IR caused the highest shift to G2/M DNA content of the treatments analysed. There were significantly greater G2/M populations observed in both PC-3 and HCT-116 PTEN-deficient cell lines compared to PTEN expressing cells (52% vs. 32%, *p* = 0.02; 48% vs. 33%, *p* = 0.04). A Caspase-Glo 3/7 assay kit was used to measure caspase-3/7 activity in the cell models (Figure 3B; Supplementary Figure S2B). Activation of caspase enzymes is a well-known indicator of early apoptosis. In both PC-3 and HCT-116 models, there was significantly increased caspase 3/7 activity in the PTEN-deficient cells when compared to PTEN expressing cells 48 h following KU-60019 (*p* ≤ 0.05; *p* ≤ 0.05) or IR (*p* ≤ 0.05; *p* ≤ 0.05) and a highly significant increase following combined KU-60019 + IR treatment (*p* ≤ 0.001; *p* ≤ 0.01).

### 2.4. Elevated Levels of DSBs in PTEN-Deficient Cells Mediated by Increased ROS

**Figure 4:**
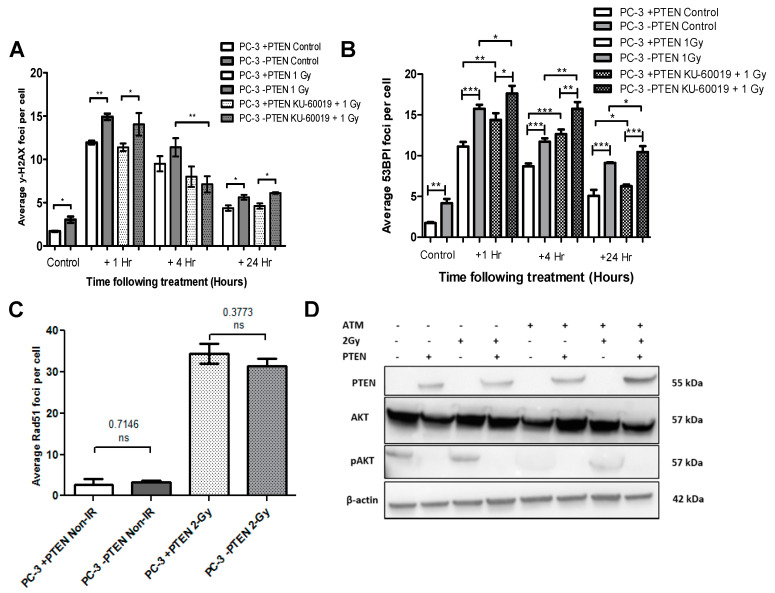
Dependency of DNA damage yields on PTEN status and combinations of radiation with KU-60019. (**A**,**B**) Levels of DNA damage in PC-3 PTEN isogenic cell models following KU-60019 and IR alone or in combination. Mean γ-H2AX and 53BP1 foci per cell was plotted at 1, 4, and 24 h post-treatment with 1 Gy IR or 1 Gy + 1 μM KU-60019. Differences between two groups were compared by using a two-sample student’s *t*-test (one asterisk (*), *p* < 0.05; two asterisks (**), *p* < 0.01; three asterisks (***), *p* < 0.001). (**C**) Mean Rad51 foci per cell in PC-3 PTEN isogenic cell models basally and 1 h following 2 Gy ionising radiation. Differences between th two groups were compared by using a two-sample student’s *t*-test. NS denotes a non-significant difference. (**D**) Western blot analysis showing basal expression levels of PTEN, Akt, and pAKT in PC-3 +PTEN and PC-3 –PTEN models.

To quantify PTEN’s effect on levels of DNA damage basally and in response to treatment with IR and KU-60019 alone and in combination, PC-3 and HCT-116 PTEN isogenic models were immunostained and counted for the phosphorylated histone γH2AX and 53BP1, sensitive determinants of DSB formation that accumulate as foci at break sites (Figure 4A,B; Supplementary Figure S4).

For the PC-3 PTEN-deficient untreated control cells, there were significantly higher mean foci per cell compared to PTEN expressing cells using both the γ-H2AX (*p* ≤ 0.05) (Figure 4A) and 53BP1 assay (*p* ≤ 0.01) (Figure 4B). Similarly, untreated HCT-116 PTEN-deficient cells had a greater quantity of foci in comparison to their WT counterpart (*p* ≤ 0.05) (Supplementary Figure S4A). Following 1 Gy radiation exposure, there was a significant increase in DNA damage that peaked at 1 h in each cell model and fell as time after treatment increased. At 24 h, foci levels remained slightly above those of non-irradiated controls, in each cell line. At 1 h and 24 h, PC-3 and HCT-116 PTEN-deficient cells exhibited higher γ-H2AX foci levels in comparison to the PTEN expressing cells (1 h for both cell models *p* ≤ 0.01; 24 h for both cell models *p* ≤ 0.05). This was consistent at 4 h in the HCT-116 cells (*p* ≤ 0.05), however, there was no significant difference in the PC-3 +PTEN and −PTEN cells at the 4 h time point. A similar trend was observed with the 53BP1 assay whereby, PC-3 PTEN-deficient cells had significantly higher mean foci in comparison to PTEN expressing cells at 1 h, 4 h and 24 h after IR (*p* ≤ 0.001; *p* ≤ 0.001; *p* ≤ 0.001).

Treatment with KU-60019 in combination with IR similarly caused significant DNA damage relative to the untreated controls. There was a significant reduction in γH2AX mean foci per cell following combination treatment compared to IR alone in PC-3 −PTEN cells at 4 h (*p* ≤ 0.01) and HCT-116 −PTEN cells at 1 and 4 h (*p* ≤ 0.01; *p* ≤ 0.05). No differences were observed in PTEN-expressing cells. However, with the 53BP1 assay, there was a statistically significant increase in mean foci per cell across all time points following combined treatment in both PC-3 PTEN-deficient (*p* ≤ 0.05; *p* ≤ 0.01; *p* ≤ 0.05) and expressing cells (*p* ≤ 0.01; *p* ≤ 0.001; *p* ≤ 0.05) in comparison to IR alone. Following combination treatment, PTEN loss again correlated with elevated γ-H2AX DNA damage; seen in the PC-3 cells at 1 and 24 h (*p* ≤ 0.05; *p* ≤ 0.05) and the HCT-116 cells at 4 and 24 h (*p* ≤ 0.05; *p* ≤ 0.05). A similar response was observed with the 53BP1 assay whereby, PC-3 PTEN-deficient cells had significantly greater mean foci per cell after combination of KU-60019 and IR in comparison to PTEN expressing cells at all time points (*p* ≤ 0.05; *p* ≤ 0.01; *p* ≤ 0.05). 

Overall, KU-60019 had very little effect on the mean γ-H2AX foci per cell, however, at the 1 h and 24 h time points, the elevated number of foci present in the PC-3 and HCT-116 PTEN-deficient cells controls compared to PTEN expressing cells was diminished (*p* ≤ 0.05; *p* ≤ 0.05) (data not shown). The associated immunofluorescence images of PC-3 and HCT-116 cells are presented in Supplementary Figure S5A,B, respectively.

To determine if an increase in DNA damage in PTEN-deficient cells was a result of defective DSB repair, mean Rad51 foci per cell in PC-3 and HCT-116 PTEN isogenic models were determined basally and following 2 Gy irradiation (Figure 4C; Supplementary Figure S4B). In the untreated control cells, there was no association between PTEN expression and Rad51 expression, in either cell model. Similarly, there was an equal increase in Rad51 foci following exposure to 2 Gy IR.

**Figure 5:**
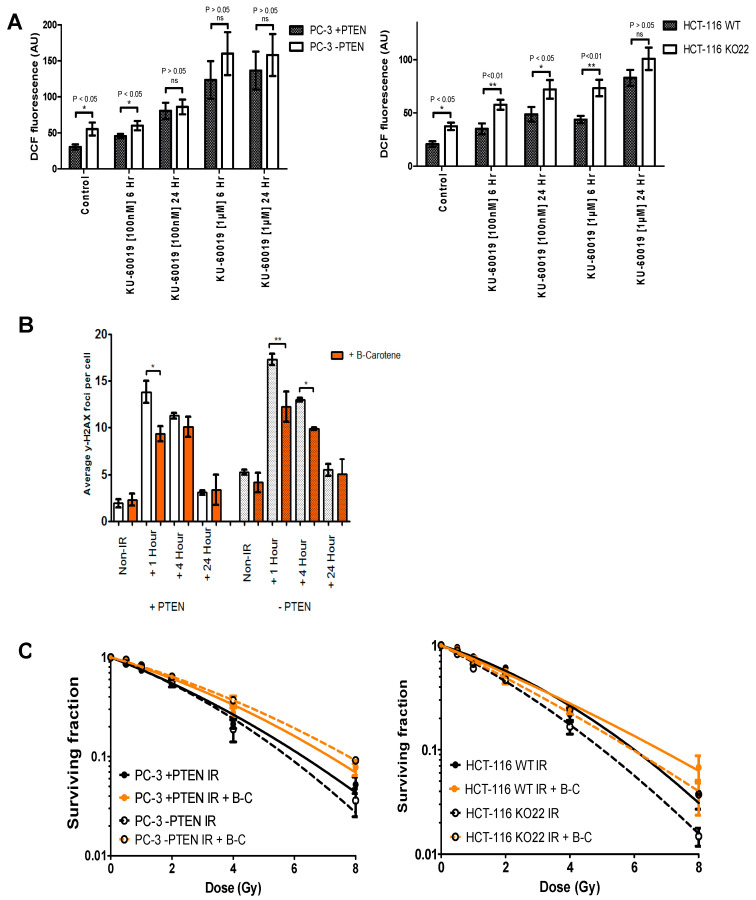
Role of reactive oxygen species and its dependency on PTEN status and combinations of radiation with KU-60019. (**A**) Quantification of reactive oxygen species (ROS) levels in PTEN isogenic cell models following KU-60019 treatment. ROS levels in PC-3 and HCT-116 +PTEN and −PTEN cells were measured basally and following treatment with 100 nM or 1 μM KU-60019. Measurements were performed by DCF-DA fluorescence assay 6 and 24 h post-treatment with the mean fluorescence levels for each group shown. AU, Arbitrary units. Differences between two groups were compared by using a two-sample student’s *t*-test. (**B**) Mean γ-H2AX foci per cell for PC-3 +PTEN and −PTEN cells 1, 4, and 24 h post-treatment with 1 Gy IR or 1 Gy IR following beta-carotene exposure. Asterisks indicate the level of statistical significance (one asterisk (*), *p* < 0.05; two asterisks (**), *p* < 0.01). (**C**) Clonogenic survival curves showing the effect of beta-carotene on radiosensitivity of +PTEN (●) and −PTEN (■) HCT-116 and PC-3 cell models compared to controls (●) and (■) respectively. Cell media was treated with beta-carotene 2 h before they were irradiated with a single radiation dose of 0.5, 1, 2, 4, or 8 Gy.

Indications from previous work showing elevated generation of ROS in PTEN-deficient cells and a subsequent increased activation of ATM [10]. Observations of significantly increased ROS generation and DNA damage in PTEN-deficient PC-3 and HCT-116 cells (Figure 5A), led to the investigation of a potential link between the two and whether this phenotype could be rescued with the use of beta-carotene, a powerful antioxidant, and free radical scavenger. The addition of beta-carotene prior to irradiation resulted in a significant reduction in mean γ-H2AX foci in PC-3 +PTEN cells at 1 h and a highly significant reduction in mean γ-H2AX foci in PC-3 −PTEN 1 h post-treatment (Figure 5B). Immunofluorescence images of PC-3 +PTEN and −PTEN cells stained for DAPI and γ-H2AX, 1, 4, and 24 h following treatment with 1 Gy IR alone or with beta-carotene are also shown. Clonogenic survival data for PC3 and HCT-116 PTEN isogenic models was generated following treatment with radiation alone or 1 h following beta-carotene exposure (Figure 5C). The addition of beta-carotene to cell media prior to irradiation produced a radioprotective effect and reduced the effect of PTEN on radiation response in both cell models.

### 2.5. ATM Inhibitor KU-60019 in Combination with Ionising Radiation Has Enhanced Anti-Tumour Activity in PTEN-Deficient Xenografts

The in vivo radiosensitising effects of KU-60019 on PTEN-deficiency were investigated in the PTEN Tet-inducible PC-3 xenograft model. Animals were treated with vehicle, KU-60019 alone (5 days × 100 mg/kg), IR alone (2 Gy), or combined KU-60019 + IR (5 days × 100 mg/kg + 2 Gy).

**Figure 6:**
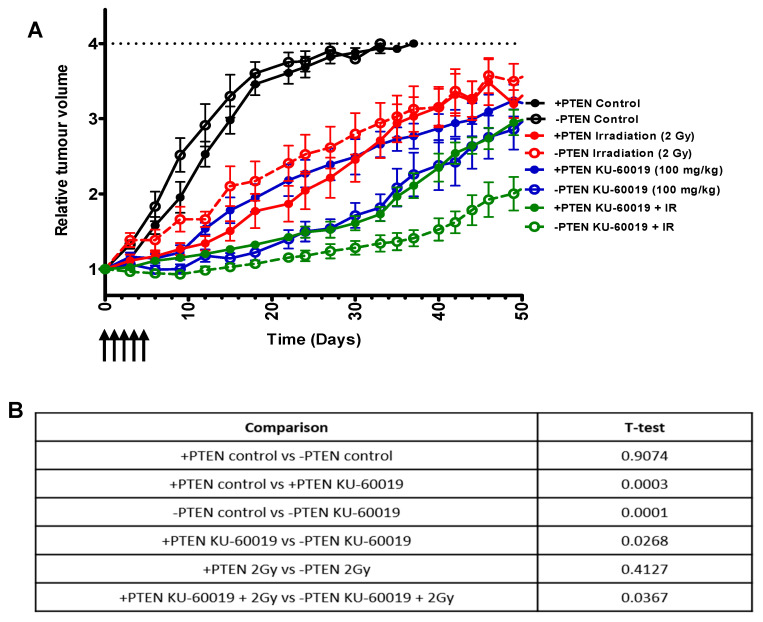

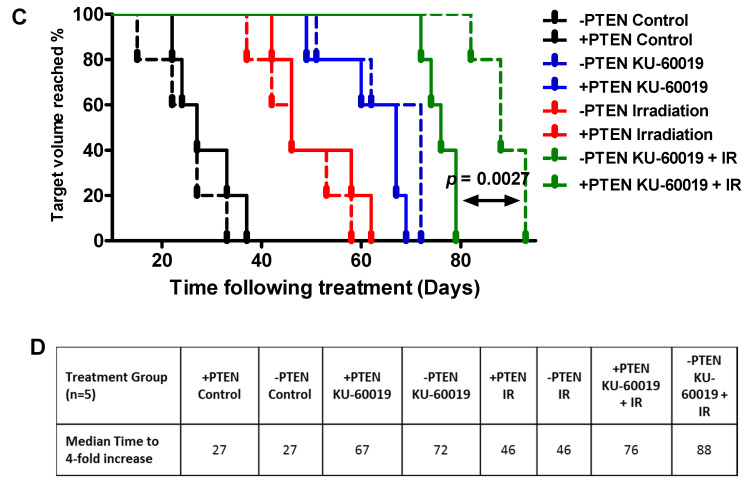
Anti-tumor response to combinations of radiation with KU-60019 and its dependency on PTEN status. (**A**) Relative tumour volume curves for PC-3 +PTEN and −PTEN subcutaneous xenografts following treatment with KU-60019, IR, or combined KU-60019 + IR treatment. Mean relative tumour volume is plotted against time from initial treatment. KU-60019 was administered at 100 mg/kg for 5 consecutive days when tumours reached 100 mm^3^. Irradiation of tumours was performed as a single dose fraction of 2 Gy. Data represent the mean ± SD of 6 mice in each group. (**B**) Statistical analysis (T-test) between different tumour volumes at day 30. (**C**) Kaplan–Meier graph (percentage animals at target volume plotted against time) for treatment groups in A. Significance between treatments was analysed by the log-rank test (Mantel–Cox). *p* = <0.05 is considered statistically significant. (**D**) Median survival (days to reach 4-fold increase in initial tumour volume) for xenograft treatment groups.

Radiotherapy alone extended the median time to 4-fold increase in median tumour volume equally between the +PTEN and −PTEN tumours (from 27 to 46 days) (Figure 6A–C). Consistent with our previous report [10] a greater tumour growth delay was initially observed in −PTEN tumours compared to +PTEN tumours following single agent KU-60019 treatment. At day 30 there is a significant difference in the +PTEN and −PTEN versus added KU-60019 which is also maintained when radiation is combined (Figure 6D). However, on discontinuation of KU-60019 the −PTEN tumour eventually recovered growth and there was no significant difference in time to reach a 4-fold increase in size (67 days vs. 72 days) (Figure 6A–C). When 5 days of KU-60019 was combined with radiotherapy a significantly longer time to reach 4-fold increase in tumour size compared to +PTEN tumours in the combination treatment group was observed (76 days vs. 88 days *p* = 0.0027) (Figure 6A–C) demonstrating the synergistic effects of these treatments. Importantly, KU-60019 in combination with 2 Gy IR was well tolerated, with no adverse effects or significant changes in body weight noted (Supplementary Figure S6A).

## 3. Discussion

PTEN loss has previously been reported to be prognostic for outcome following radiotherapy [4,15]. Clonogenic survival data from the present study (Figure 1) demonstrated the role of PTEN in sensitivity to ionising radiation is both complex and context-dependent. Deletion of PTEN in HCT-116 cells increased sensitivity to radiation, compared to wildtypes. This observation is consistent with previously reported data using DU145, HCT-116, and H-1299 knockout models [8,9,16]. Similarly, overexpression of PTEN in Hek293 produced similar radioresistance [17].

In contrast, PTEN overexpression in PC-3 cells did not alter radiosensitivity. Inducible reconstitution of PTEN in PC-3 cells using GFP-PTEN expression plasmids however was reported to confer increased radioresistance [18]. Moreover, this study reported siRNA-knockdown of PTEN in DU-145 cells reduced sensitivity to IR. Several other studies have reported loss of PTEN confers radioresistance [19,20,21].

The role for PTEN in mediating cellular radiosensitivity remains unclear; however it likely involves its roles in checkpoint control [22,23], HR repair [20,24], and apoptosis [25,26]. Moreover, the hyperactivation of AKT following PTEN loss is associated with increased radioresistance [27].

In the cell models investigated here, we did not observe any impairments in checkpoint arrest (Figure 3A; Supplementary Figure S4A) or defective apoptosis induction in the absence of PTEN following irradiation (Figure 3B; Supplementary Figure S4B). Furthermore, γH2AX and Rad51 foci analysis suggested PTEN has a negligible role in HR repair of DSBs (Figure 4). We did however identify elevated oxidative damage as a potential driver of PTEN-cell radiosensitivity in the PC-3 and HCT-116 cells (Figure 5).

Previously, loss of PTEN was shown to sensitise various cancer cells to ATM inhibition [10,28]. In the present study, we have confirmed that inhibition of ATM by KU-60019 in combination with IR, is synergistically cytotoxic to PTEN-deficient cells (Figure 2), eliciting an increase in G2/M cell populations and apoptosis induction. Promisingly, this combined treatment had minimal effect on PTEN wild-type RWPE-1 normal cells. Furthermore, KU-60019 in combination with IR significantly increased PTEN deficient tumour control compared to +PTEN tumours, with low toxicity.

We speculate that PTEN-deficient cells are sensitised to ATM inhibition as a result of elevated endogenous levels of DNA damage [10], with additional DNA-damage induced by IR leading to a lethal accumulation of genomic damage. Several previous studies have similarly reported high endogenous levels of DNA damage in PTEN-deficient cells [24,29].

We further speculate that these elevated levels of DNA damage are induced by high levels of ROS, with antioxidant treatment reducing IR-induced γ-H2AX foci (Figure 5). More work is required to determine the cause of high ROS levels, however, one potential cause is the hyperactivation of AKT, known to drive cellular metabolism, of which ROS are a by-product. Independent of AKT, the loss of PTEN is reported to impede regulation of oxidative damage, with PTEN accumulating in the nucleus following oxidative stress where it associates with p53 to enhance cell cycle arrest and reduce ROS levels [30]. It has also been clearly found that selective sensitivity of PTEN-depleted cells to ATM inhibition is directly related to the actions of KU-60019 in reducing phosphorylation of AKT (elevated in cells lacking PTEN) at ser473 [31].

Only recently have ATM inhibitors started to be tested in clinical trials, (NCT02588105) building on several promising in vivo studies using ATM inhibitors [32,33,34]. Further investigation incorporating PTEN heterozygous knockout orthotopic mouse models (more accurately reflecting human prostate cancer) is required to determine the efficacy of this new treatment strategy. Moreover, these investigations should aim to utilise SARRP (Small Animal Radiation Research Platform) to enable conformal irradiation of murine tumours, closely mimicking current clinical RT [35,36].

## 4. Materials and Methods

### 4.1. Small-Molecule Inhibitor

KU-60019, previously validated as a potent and selective inhibitor of ATM kinase [31], was purchased from Tocris (Bristol, UK). For in vitro work, KU-60019 was prepared as 10 mM stock solutions in DMSO and stored at −20 °C. For in vivo studies, KU-60019 was dissolved in Hot Rod Chemistry (HRC) Rapid Formulation 6 (Pharmatek Laboratories, San Diego, CA, USA).

### 4.2. Cell Lines

All cell lines were sourced from the American Tissue Culture Collection (ATCC, Manassas, Virginia, USA). HCT-116 cells lacking wild-type PTEN have been described previously and were licensed from Georgetown University [37]. The PC-3 PTEN-inducible cell lines have previously been described [38]. PC3-PTEN cells are PTEN null wild-type cells. Normal prostate epithelial RWPE-1 cells were purchased from ATCC. All cells were routinely screened and found to be free of mycoplasma. Western blot validation of cell models is shown in [10].

### 4.3. Radiation Treatment

IR was delivered using 225 kVp X-rays generated using an X-rad 225 irradiation system (Precision X-RAY Inc, North Branford, CT, USA). A constant dose rate of 0.55 Gy/min was used.

### 4.4. Clonogenic Cell Survival Assays

Cells were seeded into 6-well plates (Nunc, UK) and incubated for 24 h to adhere to the base of the wells. Irradiation exposures were then performed. Plating density was adjusted depending on cell model colony sizes and dose of treatment exposure. Plates were exposed to a uniform field dose, with survival determined at doses of 0.5, 1, 2, 4, and 8 Gy. Following treatment, cells were left to proliferate for 7–10 days, then fixed and stained with 2% crystal violet in 80% methanol. Colony counts were performed manually. Colonies greater than 50 cells were scored as representing surviving cells. Surviving fractions were calculated by dividing the plating efficiency for the specified dose divided by the plating efficiency of untreated cells. Radiation treatment survival curves were fitted to the linear-quadratic model formula [S = exp(−αD − βD2)]. Mean survival fractions at 2 Gy (SF2) and 4 Gy (SF4) were also obtained for each cell model and used to calculate the radiation sensitisation enhancement ratio (SER) of PTEN expression. An SER greater than 1 indicates enhancement of radiosensitivity and, similarly, an SER below the value of 1 suggests radioresistance effects. Combination treatment survival curves were plotted and fitted using a non-linear regression and analysed with Prism 5.0 software (Graphpad Software, San Diego, CA, USA). The radiation enhancement ratio (RER) was also measured; calculated by dividing the mean survival fraction at KU-60019 [1 µM] by that for the combined therapy [1 µM + 2 Gy]. Similarly, the dose enhancement ratio (DER) of combined IR + KU-60019 [LC_50_ −PTEN] was calculated by dividing the dose (Gy) required to kill 75% of cells (SF_0.25_) for radiation alone by the SF_0.25_ dose for radiation + KU-60019.

### 4.5. Flow Cytometry Analysis of Cell Cycle

Following treatment with 2 Gy IR, 1 µM KU-60019, or combination for 48 h, cells were collected and fixed overnight with 70% ethanol (−20 °C). At the time of analysis, cells were incubated with 100 µg/mL RNase A for 30 min at 37 °C. Following incubation, propidium iodide was added at RT. Flow cytometry was then performed on a BD-LSR II (BD Biosciences, Oxford, UK).

### 4.6. Apoptosis Analysis

Cells were seeded into white-walled 96-well plates. The following day, cells were exposed to a single 4 Gy dose of IR and 1µM KU-60019, separately or in combination. Drug vehicle and sham irradiated controls, and a blank control well with assay reagent were also assayed. In the combination treatments, cells were exposed to IR, 1 h immediately after KU-60019 treatment. At 48 h post-treatment, the caspase-glo 3/7 assay (Promega, Southampton, UK) was performed to measure caspase-3 and -7 activities in the treated cells. Fluorescence was measured using a fluorescence microplate reader (Bio-Tek, Bedfordshire, UK).

### 4.7. Reactive Oxygen Species Detection

Analysis of oxidative species was carried out using a dichloro-dihydro-fluorescein diacetate (DCF-DA) assay kit (Life Technologies, Paisley, UK). Cells were plated into 96-well plates and incubated overnight to adhere. The following day, cells were treated with 100 nM or 1 µM KU-60019. Fluorescence was measured for each time point at a 485 nm excitation wavelength and a 527 nm emission wavelength using a microplate reader. The background fluorescence from cell-free controls was subtracted.

### 4.8. Immunofluorescence

Cells were grown on coverslips inside 6-well plates. At specified times following treatment, cell media was aspirated and cells gently washed with PBS. Cells were fixed with 4% paraformaldehyde for 15 min at RT, then permeabilised using 0.5% Triton X-100 for 20 min at 4 °C. Cells were blocked with blocking buffer (0.1% Triton X-100, 3% BSA in PBS) for 60 min at 4 °C. Next, cells were incubated in γH2AX, Rad51 primary antibody (Abcam, Cambridge, UK) or anti53BP1 (Novus Biologicals, Centennial, CO, USA) diluted in blocking buffer (1:500) overnight at 4 °C. Following washing, Alexafluor 488-labelled or Alexaflour 568-labelled secondary antibodies (Life Technologies, UK) diluted in blocking buffer (1:500) was added for 30 min at RT. Nuclei were then stained by incubation with DAPI (4, 6-diamidino-2-phenylindole) for 5 min at RT in the dark. Mounting of specimens required the coverslips to be removed from the well with care and dipped in fresh PBS. Any excess liquid was blotted off and 2 drops of mounting media was added to the coverslip. The coverslips were placed (cells facing downwards) onto 900 microscope slides (Menzel Glaser, Germany). Stained slides were viewed in dark conditions using a Zeiss Axiovert 200 M microscope (Carl Zeiss MicroImaging, LLC, White Plains, NY, USA). For each treatment condition, γH2AX foci were determined in at least 50 cells.

### 4.9. In Vivo Xenograft Studies

Subcutaneous xenograft tumours derived from the PC-3 PTEN tetracycline-inducible cell model and were grown in male Fox Chase SCID (Severe Combined Immunodeficiency) mice (Charles River Laboratories, Oxford, UK). PC-3 PTEN tetracycline-inducible cells were grown in monolayer culture and harvested using trypsin on the day of implantation. 100 µL of cell suspension (3 × 10^6^ cells) was injected subcutaneously into the right flank of each mouse using a sterile syringe and 21G needle. Simultaneously, mice were subcutaneously implanted with transponders for unique identification (Avid Identification Systems, Norco, CA, USA).

Xenograft tumour generation was monitored using volumetric measurements with external callipers. In order to assess tumour volume by external callipers, the greatest longitudinal diameter (length), the greatest transverse diameter (width) and the greatest vertical diameter (breadth) were measured and the geometric mean diameter (GMD) calculated. Tumour volume estimates could then be derived.

$$GMD=\;\sqrt[{3}]{\left(L\times B\times H\right)}\;r=\frac{GMD}{2}\;volume=\left(\frac{4}{3}\right)\times \left(\pi \times r^{3}\right)$$

When tumour volumes reached approximately 100 mm^3^, mice were randomly assigned into treatment groups. PTEN was induced in the specified PTEN+/+ tumours by the oral gavage administration of 100 µL doxycycline [16 mg/kg] and repeated every 48 h for the duration of the experiment. KU-60019 and drug vehicle were administered at 100 mg/kg daily, in a volume of 200 µL for 5 or 10 consecutive days. Irradiation of tumours was performed by immobilising mice in a universal rodent restrainer (Harvard Apparatus, Kent, UK). In combination treatment groups, irradiation was performed 1 h immediately following the final KU-60019 administrations. Each treatment group contained 5 animals. All experiments were carried out in accordance with the local ethical and Home Office Regulations (ASPA19/project license 2945) and designed in accordance with the Scientific Procedures Act (1986) and the 2010 Guidelines for the welfare and use of animals in cancer research [39].

### 4.10. Statistical Analysis

Two-tailed student’s *t*-test was used to determine statistical significance between means. Estimates of time to tumour formation were determined using Kaplan–Meier methods and groups compared using the log-rank test.

## 5. Conclusions

In conclusion, the results of this study identified potent radiation sensitising effects of KU-60019 on PTEN-deficient cells and xenograft tumours. Our results suggest this sensitivity is mediated by elevated oxidative damage rather than the cells’ capacities to repair DNA damage. Inhibiting ATM-mediated regulation of oxidative damage in these cells harbouring elevated endogenous DNA damage, further contributed to by IR-induced DSBs, creates an accumulative DNA damage burden that is lethal. We speculate elevated levels of oxidative damage in PTEN-deficient cells are driven by hyperactivation of AKT.

Elucidating PTEN loss as a therapeutic target for combined IR with pharmacological inhibition of ATM presents a promising novel treatment approach in PCa. Using gene expression profiling, it would be possible to identify patient tumours with PTEN mutations, suitable for treatment with ATM inhibitors as a neo-adjuvant or adjuvant with radiotherapy. This therapeutic strategy may help reduce the rates of biochemical failure observed in locally advanced PCa and prevent the progression to metastatic CRPC.

## Data Availability

The data presented in this study are available on request from the corresponding author.

## Supplementary Materials

The following are available online at https://www.mdpi.com/2072-6694/13/1/79/s1, Table S1: Radiobiological parameters derived from the LQ-fitted clonogenic survival data. Alpha (α) and beta (β) parameters (±SEM) are representative of the linear and quadratic components of the LQ-fitted curves, respectively. SF2 and SF4 represent survival fractions at doses 2 Gy and 4 Gy. Table S2: Radiation Sensitisation Enhancement Ratios of PTEN expression for PC-3, and HCT-116 models, derived from survival fractions at 4 Gy. Differences between +PTEN and –PTEN groups were compared by using a two-sample student’s *t*-test. Table S3: Survival values of PTEN isogenic models, derived from dose response curves following KU-60019 single agent treatment or combined KU-60019 and radiation treatment. The radiation enhancement ratios (RER) were obtained by dividing the mean surviving fraction for KU-60019 [1 μM] by the mean surviving fraction for combined KU-60019 + radiation treatment [1 μM + 2 Gy]. Also presented are the CI values for each cell type following the combined treatment at 1 μM + 2 Gy. Differences between two groups were compared by using a two-sample student’s *t*-test. Table S4: The dose enhancement ratio (DER) was calculated by dividing the dose (Gy) required to kill 75% of cells (SF0.25) for radiation alone by the SF0.25 dose for radiation + KU-60019. Differences between two groups were compared by using a two-sample student’s *t*-test. Figure S1: Clonogenic survival comparing differential response of prostate cancer cell lines following treatment with radiation alone or in combination with KU-60019. In the combined treatment groups, cells received 2 Gy ionising radiation, 1 h immediately following KU-60019 treatment. Experiments were performed in triplicate on at least three independent occasions. Error bars represent ± SEM. Differences between two groups were compared by using a two-sample student’s *t*-test (one asterisk (*), *p* < 0.05; two asterisks (**), *p* < 0.01; three asterisks (***), *p* < 0.001). Figure S2: Combination index–fraction affected plot for PTEN-isogenic models treated with KU-60019 in combination with 2 Gy ionising radiation. KU-60019 and 2 Gy IR treatments below the dashed line represent synergistic interaction. CI values were generated using Calcusyn software (Biosoft, Cambridge, UK) based on methods of Chou and Talalay. A CI value < 0.90 is indicative of synergistic interactions, CI of 0.90 to 1.0 indicates additive interactions, and CI > 1.0 indicates antagonistic interactions. Points 5 = 10 nM, 4 = 100 nM, 3 = 500 nM, 2 = 1 μM and 1 = 10 μM of KU-60019. Figure S3: (A) Cell-cycle analysis using Propidium iodide (PI) staining and detection by flow cytometry of HCT-116 WT and KO22 cell models after treatment with 1μM KU-60019 and 2 Gy IR, as single modalities and in combination. Analyses of cell cycle distributions 48 h post-treatment are shown. (B) Caspase 3/7 activity in HCT-116 WT and KO22 cell models following treatment with 1μM KU-60019 and 4 Gy IR alone or in combination. Activity was measured using a Caspase-Glo 3/7 assay kit 48 h post-treatment. Figure S4: (A) Levels of DNA damage in HCT-116 WT and KO22 cells following KU-60019 and IR alone or in combination. Mean γ-H2AX foci per cell was plotted at 1, 4 and 24 h post-treatment with 1 Gy IR or 1 Gy + 1 μM KU-60019. Differences between two groups were compared by using a two-sample student’s *t*-test (one asterisk (*), *p* < 0.05; two asterisks (**), *p* < 0.01). (B) Mean Rad51 foci per cell in HCT-116 WT and KO22 cells basally and 1 h following 2 Gy ionising radiation. Differences between two groups were compared by using a two-sample student’s *t*-test. NS denotes a non-significant difference. (C) Western blot analysis showing basal expression of total Rad51 in HCT-116 WT and KO22 cell models. Figure S5: Immunofluorescence images of (A) PC-3 and (B) HCT-116 isogenic models showing staining of phosphorylated histone H2AX foci (γ-H2AX) (Green) and 4,6- diamidino-2-phenylindole (DAPI) (Blue). Images were taken of cells at 1, 4 and 24 h post-treatment with 1μM KU-60019 and 1 Gy IR alone or in combination. Figure S6: Box plot of mean relative change in animal body weight following treatments with KU-60019, Ionising radiation and combination treatments. Figure S7 Uncropped Western Blots from Figure 4D.

## References

[1] Bonkhoff H. (2012). Factors implicated in radiation therapy failure and radiosensitization of prostate cancer. Prostate Cancer.

[2] Gray I.C., Stewart L.M., Phillips S.M., Hamilton J.A., Gray N.E., Watson G.J., Spurr N.K., Snary D. (1998). Mutation and expression analysis of the putative prostate tumour-suppressor gene PTEN. Br. J. Cancer.

[3] Zafarana G., Ishkanian A.S., Malloff C.A., Locke J.A., Sykes J., Thoms J., Lam W.L., Squire J.A., Yoshimoto M., Ramnarine V.R. (2012). Copy number alterations of c-MYC and PTEN are prognostic factors for relapse after prostate cancer radiotherapy. Cancer.

[4] Bedolla R., Prihoda T.J., Kreisberg J.I., Malik S.N., Krishnegowda N.K., Troyer D.A., Ghosh P.M. (2007). Determining risk of biochemical recurrence in prostate cancer by immunohistochemical detection of PTEN expression and Akt activation. Clin. Cancer Res..

[5] Shen M.M., Abate-Shen C. (2007). Pten inactivation and the emergence of androgen-independent prostate cancer. Cancer Res..

[6] Hou S.Q., Ouyang M., Brandmaier A., Hao H., Shen W.H. (2017). PTEN in the maintenance of genome integrity: From DNA replication to chromosome segregation. Bioessays.

[7] Mendes-Pereira A.M., Martin S.A., Brough R., McCarthy A., Taylor J.R., Kim J.S., Waldman T., Lord C.J., Ashworth A. (2009). Synthetic lethal targeting of PTEN mutant cells with PARP inhibitors. EMBO Mol. Med..

[8] Mansour W.Y., Tennstedt P., Volquardsen J., Oing C., Kluth M., Hube-Magg C., Borgmann K., Simon R., Petersen C., Dikomey E. (2018). Loss of PTEN-assisted G2/M checkpoint impedes homologous recombination repair and enhances radio-curability and PARP inhibitor treatment response in prostate cancer. Sci. Rep..

[9] Fraser M., Zhao H., Luoto K.R., Lundin C., Coackley C., Chan N., Joshua A.M., Bismar T.A., Evans A., Helleday T. (2012). PTEN deletion in prostate cancer cells does not associate with loss of RAD51 function: Implications for radiotherapy and chemotherapy. Clin. Cancer Res..

[10] McCabe N., Hanna C., Walker S.M., Gonda D., Li J., Wikstrom K., Savage K.I., Butterworth K.T., Chen C., Harkin D.P. (2015). Mechanistic Rationale to Target PTEN-Deficient Tumor Cells with Inhibitors of the DNA Damage Response Kinase ATM. Cancer Res..

[11] Barzilai A., Rotman G., Shiloh Y. (2002). ATM deficiency and oxidative stress: A new dimension of defective response to DNA damage. Dna Repair.

[12] Sun H., Lesche R., Li D.M., Liliental J., Zhang H., Gao J., Gavrilova N., Mueller B., Liu X., Wu H. (1999). PTEN modulates cell cycle progression and cell survival by regulating phosphatidylinositol 3,4,5,-trisphosphate and Akt/protein kinase B signaling pathway. Proc. Natl. Acad. Sci. USA.

[13] Radu A., Neubauer V., Akagi T., Hanafusa H., Georgescu M.M. (2003). PTEN induces cell cycle arrest by decreasing the level and nuclear localization of cyclin D1. Mol. Cell Biol..

[14] Kim S.J., Lee H.W., Baek J.H., Cho Y.H., Kang H.G., Jeong J.S., Song J., Park H.S., Chun K.H. (2016). Activation of nuclear PTEN by inhibition of Notch signaling induces G2/M cell cycle arrest in gastric cancer. Oncogene.

[15] Fontugne J., Lee D., Cantaloni C., Barbieri C.E., Caffo O., Hanspeter E., Mazzoleni G., Dalla Palma P., Rubin M.A., Fellin G. (2014). Recurrent prostate cancer genomic alterations predict response to brachytherapy treatment. Cancer Epidemiol. Biomark. Prev..

[16] Fraser M., Harding S.M., Zhao H., Coackley C., Durocher D., Bristow R.G. (2011). MRE11 promotes AKT phosphorylation in direct response to DNA double-strand breaks. Cell Cycle.

[17] Pattje W.J., Schuuring E., Mastik M.F., Slagter-Menkema L., Schrijvers M.L., Alessi S., van der Laan B.F., Roodenburg J.L., Langendijk J.A., van der Wal J.E. (2010). The phosphatase and tensin homologue deleted on chromosome 10 mediates radiosensitivity in head and neck cancer. Br. J. Cancer.

[18] Sun Y., St Clair D.K., Fang F., Warren G.W., Rangnekar V.M., Crooks P.A., St Clair W.H. (2007). The radiosensitization effect of parthenolide in prostate cancer cells is mediated by nuclear factor-kappaB inhibition and enhanced by the presence of PTEN. Mol. Cancer.

[19] Kao G.D., Jiang Z., Fernandes A.M., Gupta A.K., Maity A. (2007). Inhibition of phosphatidylinositol-3-OH kinase/Akt signaling impairs DNA repair in glioblastoma cells following ionizing radiation. J. Biol. Chem..

[20] Pappas G., Zumstein L.A., Munshi A., Hobbs M., Meyn R.E. (2007). Adenoviral-mediated PTEN expression radiosensitizes non-small cell lung cancer cells by suppressing DNA repair capacity. Cancer Gene..

[21] Christensen M., Najy A.J., Snyder M., Movilla L.S., Kim H.R. (2014). A critical role of the PTEN/PDGF signaling network for the regulation of radiosensitivity in adenocarcinoma of the prostate. Int. J. Radiat. Oncol. Biol. Phys..

[22] Puc J., Keniry M., Li H.S., Pandita T.K., Choudhury A.D., Memeo L., Mansukhani M., Murty V.V., Gaciong Z., Meek S.E. (2005). Lack of PTEN sequesters CHK1 and initiates genetic instability. Cancer Cell.

[23] Chung J.H., Ostrowski M.C., Romigh T., Minaguchi T., Waite K.A., Eng C. (2006). The ERK1/2 pathway modulates nuclear PTEN-mediated cell cycle arrest by cyclin D1 transcriptional regulation. Hum. Mol. Genet..

[24] Bassi C., Ho J., Srikumar T., Dowling R.J., Gorrini C., Miller S.J., Mak T.W., Neel B.G., Raught B., Stambolic V. (2013). Nuclear PTEN controls DNA repair and sensitivity to genotoxic stress. Science.

[25] Lehman J.A., Waning D.L., Batuello C.N., Cipriano R., Kadakia M.P., Mayo L.D. (2011). Induction of apoptotic genes by a p73-phosphatase and tensin homolog (p73-PTEN) protein complex in response to genotoxic stress. J. Biol. Chem..

[26] Lu X.X., Cao L.Y., Chen X., Xiao J., Zou Y., Chen Q. (2016). PTEN Inhibits Cell Proliferation, Promotes Cell Apoptosis, and Induces Cell Cycle Arrest via Downregulating the PI3K/AKT/hTERT Pathway in Lung Adenocarcinoma A549 Cells. Biomed. Res. Int..

[27] Toulany M., Kehlbach R., Florczak U., Sak A., Wang S., Chen J., Lobrich M., Rodemann H.P. (2008). Targeting of AKT1 enhances radiation toxicity of human tumor cells by inhibiting DNA-PKcs-dependent DNA double-strand break repair. Mol. Cancer.

[28] Li K., Yan H., Guo W., Tang M., Zhao X., Tong A., Peng Y., Li Q., Yuan Z. (2018). ATM inhibition induces synthetic lethality and enhances sensitivity of PTEN-deficient breast cancer cells to cisplatin. Exp. Cell Res..

[29] Puc J., Parsons R. (2005). PTEN loss inhibits CHK1 to cause double stranded-DNA breaks in cells. Cell Cycle.

[30] Chang C.J., Mulholland D.J., Valamehr B., Mosessian S., Sellers W.R., Wu H. (2008). PTEN nuclear localization is regulated by oxidative stress and mediates p53-dependent tumor suppression. Mol. Cell Biol..

[31] Golding S.E., Rosenberg E., Valerie N., Hussaini I., Frigerio M., Cockcroft X.F., Chong W.Y., Hummersone M., Rigoreau L., Menear K.A. (2009). Improved ATM kinase inhibitor KU-60019 radiosensitizes glioma cells, compromises insulin, AKT and ERK prosurvival signaling, and inhibits migration and invasion. Mol. Cancer.

[32] Vecchio D., Daga A., Carra E., Marubbi D., Raso A., Mascelli S., Nozza P., Garre M.L., Pitto F., Ravetti J.L. (2015). Pharmacokinetics, pharmacodynamics and efficacy on pediatric tumors of the glioma radiosensitizer KU60019. Int. J. Cancer.

[33] Riches L.C., Trinidad A.G., Hughes G., Jones G.N., Hughes A.M., Thomason A.G., Gavine P., Cui A., Ling S., Stott J. (2020). Pharmacology of the ATM Inhibitor AZD0156: Potentiation of Irradiation and Olaparib Responses Preclinically. Mol. Cance.

[34] Durant S.T., Zheng L., Wang Y., Chen K., Zhang L., Zhang T., Yang Z., Riches L., Trinidad A.G., Fok J.H.L. (2018). The brain-penetrant clinical ATM inhibitor AZD1390 radiosensitizes and improves survival of preclinical brain tumor models. Sci. Adv..

[35] Wong J., Armour E., Kazanzides P., Iordachita I., Tryggestad E., Deng H., Matinfar M., Kennedy C., Liu Z., Chan T. (2008). High-resolution, small animal radiation research platform with x-ray tomographic guidance capabilities. Int. J. Radiat. Oncol. Biol. Phys..

[36] Butterworth K.T., Prise K.M., Verhaegen F. (2015). Small animal image-guided radiotherapy: Status, considerations and potential for translational impact. Br. J. Radiol..

[37] Lee C., Kim J.S., Waldman T. (2004). PTEN gene targeting reveals a radiation-induced size checkpoint in human cancer cells. Cancer Res..

[38] Maxwell P.J., Coulter J., Walker S.M., McKechnie M., Neisen J., McCabe N., Kennedy R.D., Salto-Tellez M., Albanese C., Waugh D.J. (2013). Potentiation of inflammatory CXCL8 signalling sustains cell survival in PTEN-deficient prostate carcinoma. Eur. Urol..

[39] Workman P., Aboagye E.O., Balkwill F., Balmain A., Bruder G., Chaplin D.J., Double J.A., Everitt J., Farningham D.A., Glennie M.J. (2010). Guidelines for the welfare and use of animals in cancer research. Br. J. Cancer.

