# Supplementary material for: ATM Kinase Inhibition Preferentially Sensitises PTEN-Deficient Prostate Tumour Cells to Ionising Radiation
Source: Cancers (Basel). 2020 Dec 30;13(1):79. doi: 10.3390/cancers13010079 (PMC7794981; doi:10.3390/cancers13010079)

Supplementary data

Supp Table 1 & 2.

	**PC-3 +PTEN**	**PC-3 -PTEN**	**HCT-116 WT**	**HCT-116 KO22**	**RWPE-1**
α	0.32±0.04	0.27±0.07	0.28±0.05	0.36±0.09	0.46±0.08
β	0.007±0.01	0.019±0.01	0.017±0.01	0.021±0.01	0.011±0.01
SF2	0.52	0.58	0.54	0.45	0.38
SF4	0.23	0.23	0.25	0.17	0.13

Radiation Sensitisation Enhancement Ratio of PTEN expression

	**PC-3**	**HCT-116**
SER	0.92 (p = 0.41)	0.68 (p = 0.02)

Supplementary data

Supp Figure 1

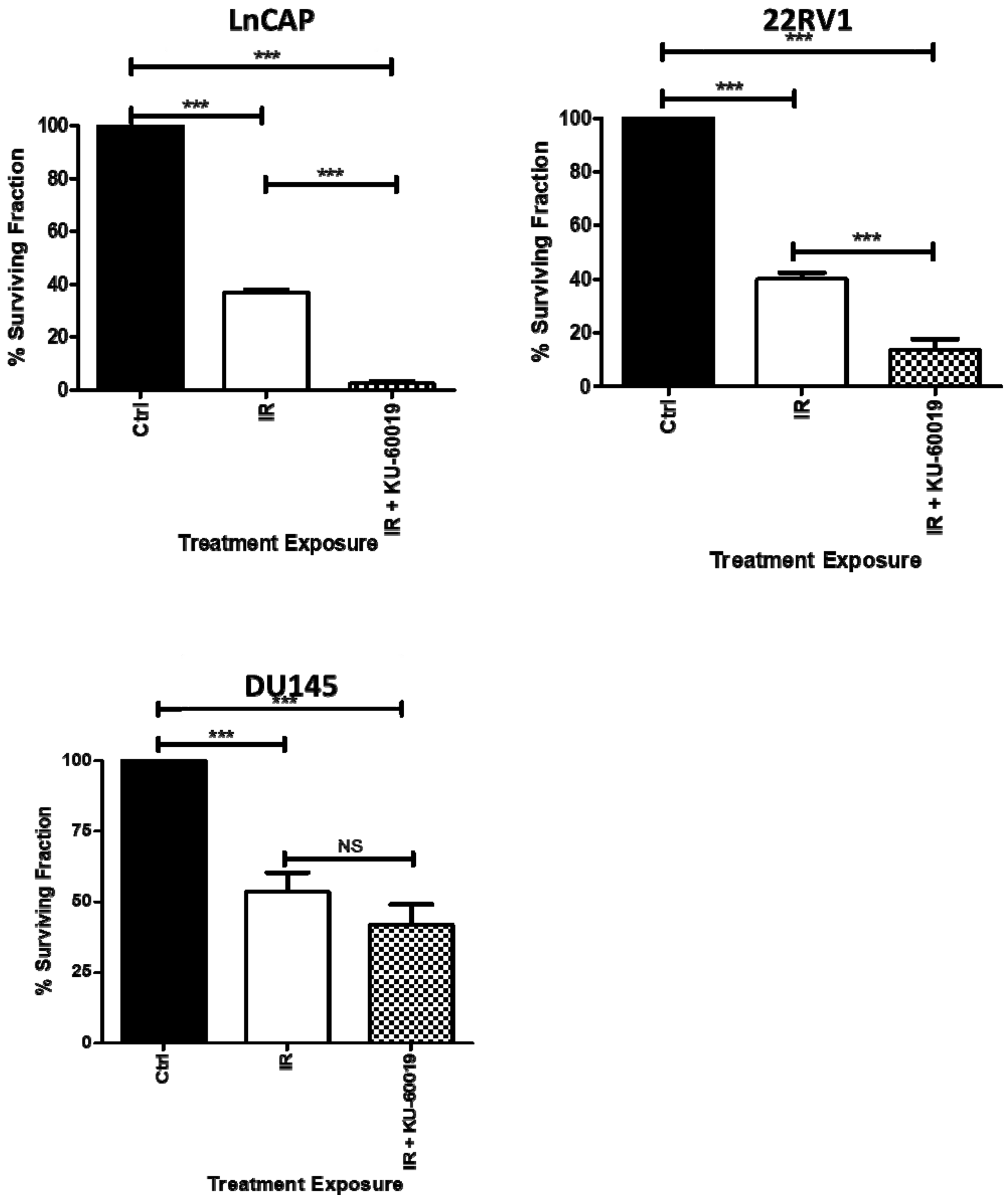

Supplementary data

Supp Figure 2

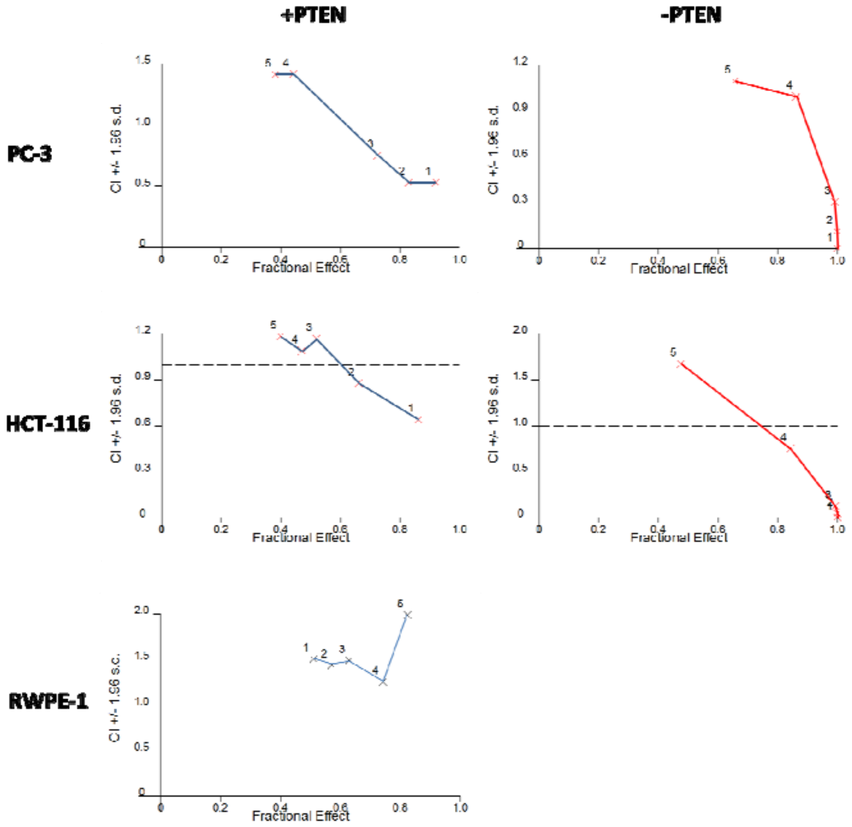

Supplementary data

Supp Table 3

	**PC-3 +PTEN**	**PC-3 -PTEN**	**HCT-116 WT**	**HCT-116 KO22**	**RWPE1**
SF (1μM)	0.33	0.03	0.70	0.19	0.38
SF (2-Gy + 1μM)	0.17	0.01	0.34	0.16	0.26
RER	1.94	3.00	2.06	1.13	1.47
Combination Index (2-Gy + 1μM)	0.535	0.115	0.88	0.13	1.25

Supplementary data

Supp Table 4

	**PC-3 +PTEN**	**PC-3 -PTEN**	**HCT-116 WT**	**HCT-116 KO22**
$SF_{0.25}$ Dose (IR)	4.08	3.82	3.94	3.23
$SF_{0.25}$ Dose (IR + [LC_{50} –PTEN])	4.41	2.33	3.75	2.33
DER	0.93 (p = 0.03)	1.64 (p = 0.01)	1.05 (p = 0.019)	1.39 (p = 0.02)

Supplementary data

Supp Figure 3

A

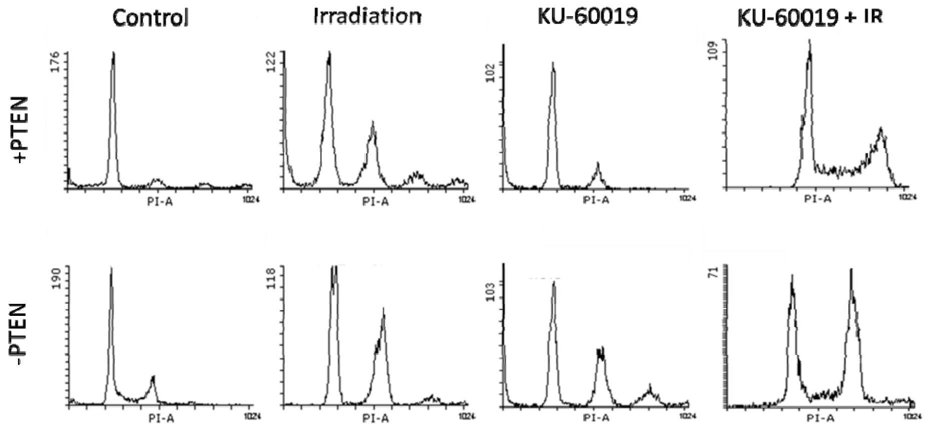

	+PTEN Control	-PTEN Control	+PTEN IR	-PTEN IR	+PTEN KU-60019	-PTEN KU-60019	+PTEN KU-60019 + IR	-PTEN KU-60019 + IR
G1	72	50	58	47	56	42	49	40
S	7	19	5	9	12	12	10	5
G2/M	14	27	27	36	27	39	33	48

B

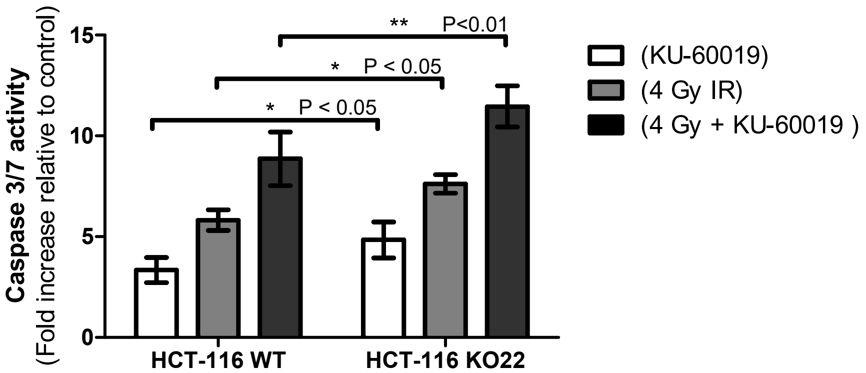

Supplementary data

Supp Figure 4

A

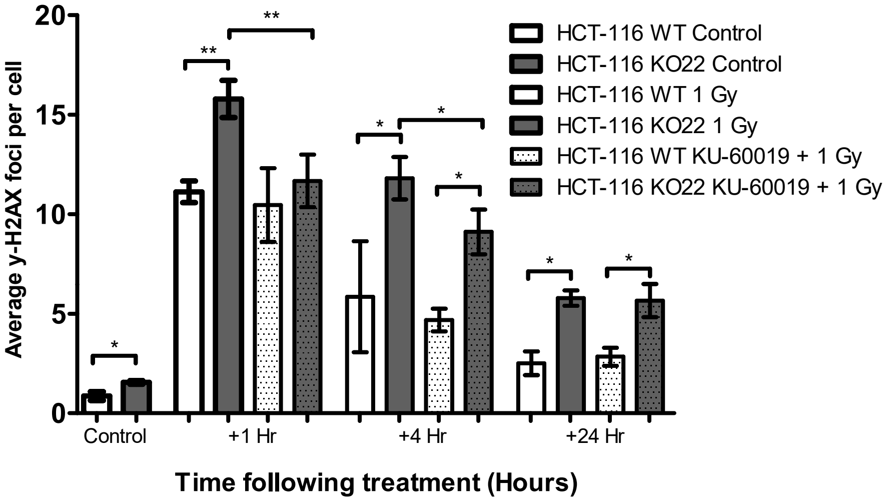

B

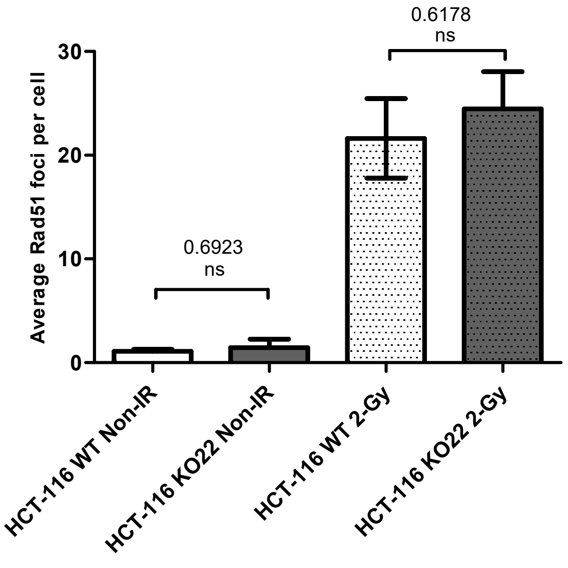

C

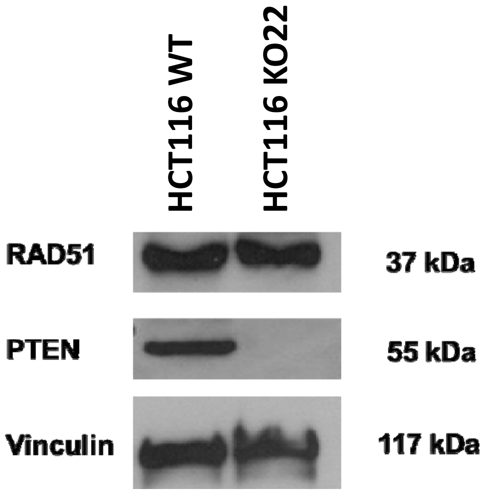

Supplementary data

Supp Figure 5

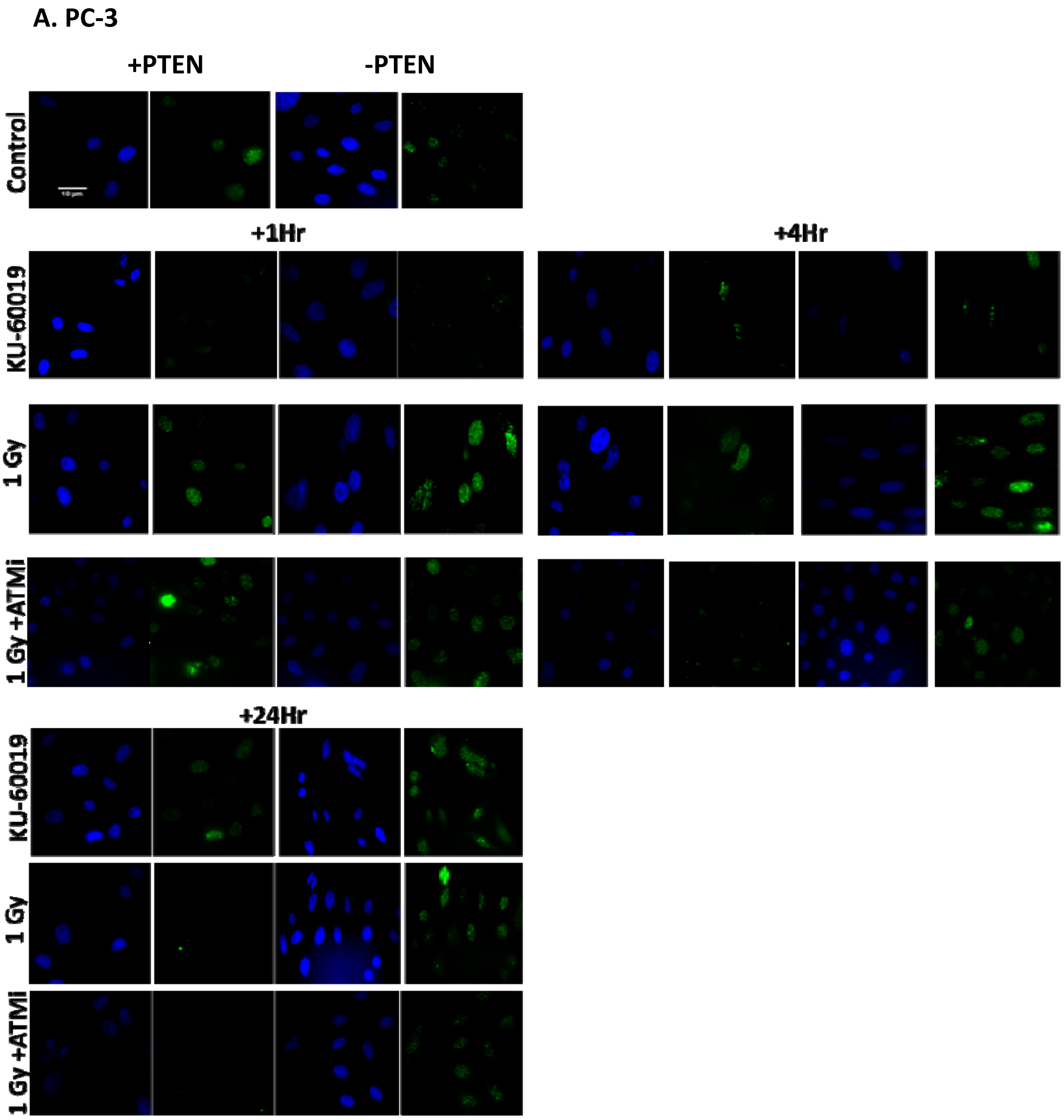

Supplementary data

Supp Figure 5

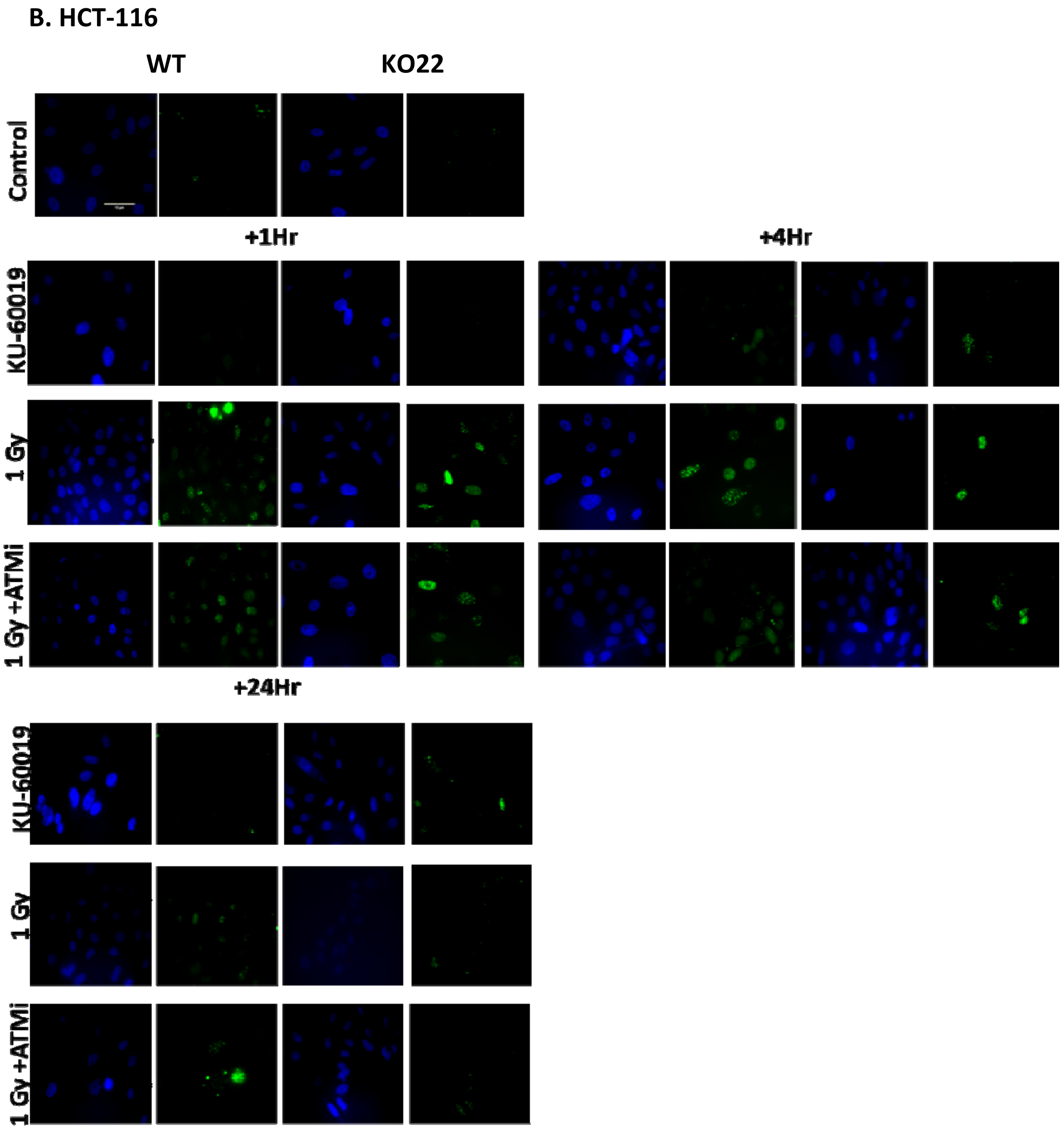

Supplementary data

Supp Figure 6

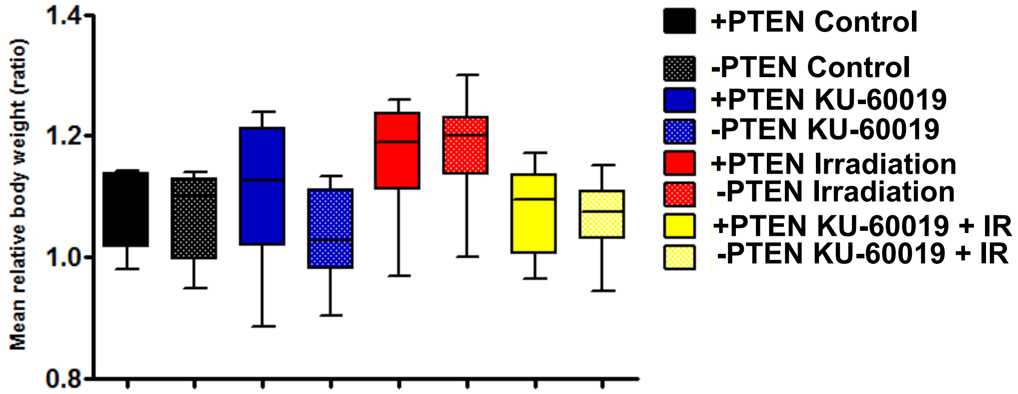

Supplementary data

Supp Figure 7

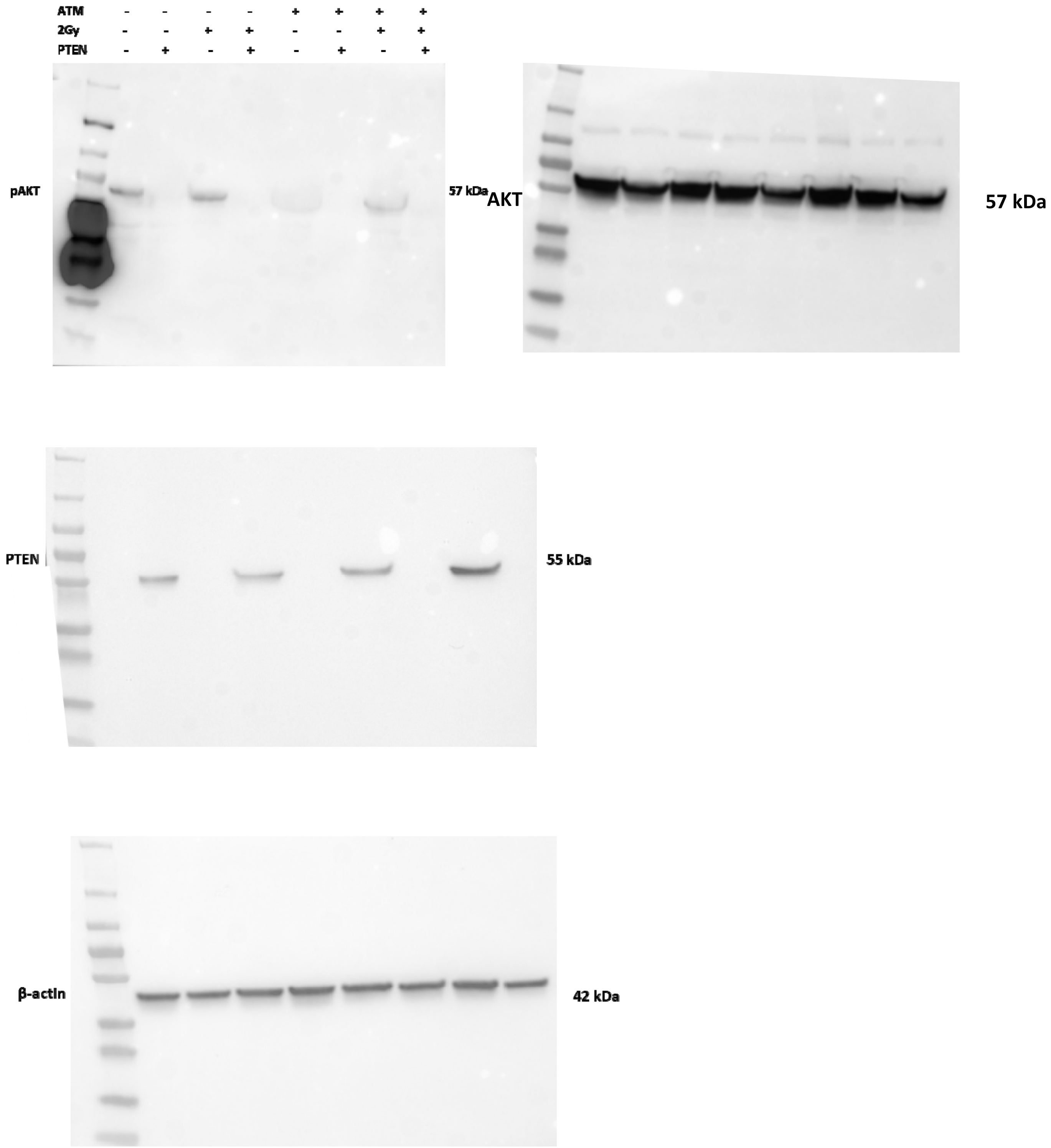

Supplement: Supplementary file 1 [file cancers-13-00079-s001.pdf]
